# The burden of acute pain in the U.S. in the wake of the opioid crisis

**DOI:** 10.3389/fpain.2025.1642035

**Published:** 2025-10-07

**Authors:** James C. Hackworth, John E. Schneider, Maggie Do Valle, David Fam, Charles Argoff, Emanuela Offidani, Jim Potenziano

**Affiliations:** ^1^ Tris Pharma, Monmouth Junction, NJ, United States; ^2^ Avalon Health Economics, Morristown, NJ | Miami, FL, United States; ^3^ Albany Medical College, Albany, NY, United States

**Keywords:** acute pain, opioid use disorder (OUD), pain management, undertreatment, chronification of pain, healthcare costs

## Abstract

The prevalence of acute pain has grown substantially over the past two decades, due primarily to more surgeries, an aging population, and the rapid growth in the prevalence of metabolic disease. Although opioids are often the only effective treatment for many types of acute pain, especially severe acute pain, their use, even over a short period of time, comes with substantial risks of dependence, misuse, and diversion. Moreover, a large fraction of the patients currently suffering from opioid use disorder and those dying from opioid overdoses had their first exposure as pain patients. Conversely, refraining from using opioids in cases where other treatment options are ineffective creates a different set of risks. This potential undertreatment of acute pain, especially severe acute pain, increases the risk of acute pain transitioning to chronic pain. The use of opioids to treat acute pain and the ineffective treatment of acute pain have important implications for population health and health care costs.

## 1 Introduction

Managing acute pain in the United States remains a critical clinical and public health challenge. Since 1998, the prevalence of pain among U.S. adults has increased by 25% (1). Pain is reported in nearly 80% of emergency department (ED) visits in the U.S., with a median reported pain intensity score of 8 out of 10 on numeric rating scales (2). The prevalence of moderate to severe acute pain continues to rise, often stemming from post-operative procedures but also associated with the rising prevalence of metabolic disorders and an aging population. The rising prevalence of pain has imposed a significant burden on both patients and healthcare systems. Moreover, individuals with poorly treated acute pain are at greater risk for developing chronic pain, which further widens the treatment gap. Although opioids are an effective treatment for acute pain, especially severe acute pain, providers and patients have, understandably, approached opioid-based treatments with trepidation, and clinical practice guidelines have not completely resolved these apprehensions in the wake of the opioid crisis in the U.S. Consequently, the aftermath of the opioid crisis has resulted in a greater risk of the undertreatment of acute pain.

Acute pain is defined by the International Association for the Study of Pain (IASP) as pain that “happens suddenly, starts out sharp or intense, and serves as a warning sign of disease or threat to the body. It is caused by injury, surgery, illness, trauma, or painful medical procedures and generally lasts from a few minutes to less than six months.” (3). The IASP definition further notes that “acute pain usually disappears whenever the underlying cause is treated or healed.” Examples of acute pain include pain associated with surgery, musculoskeletal injuries (e.g., broken bones, sprains, and strains), dental procedures, soft tissue injuries (e.g., cuts, puncture wounds, burns), blunt force trauma, labor and childbirth, and headaches (4). Subacute pain, as labeled by the U.S. Centers for Disease Control and Prevention (CDC), is generally defined as pain lasting one to three months. However, the literature focused on subacute pain does not consistently apply this threshold, with some sources classifying acute pain as episodes of six weeks or less in duration, and subacute with episodes of seven to twelve weeks. While the duration of subacute pain may in some cases overlap with that of acute pain, it is generally recognized as a separate clinical stage, whereas chronic pain generally extends beyond the three-month mark (5, 6).

Aside from different durations of pain there are different pain conditions, e.g., neuropathic pain, visceral pain, inflammatory pain, etc. that may respond differently to treatment as a result of their underlying mechanisms and characteristics (7). For example, neuropathic pain is usually characterized as damage to the nerve, often leading to burning, aching, or stabbing sensations and can either be acute or chronic. In contrast, inflammatory pain is typically caused by inflammation in tissues like joints or muscles, and tends to be relatively more localized, examples of which include musculoskeletal pain, trauma-related pain, dental pain, and post-surgical pain (8). Understanding these causal pathways is critical to improving patient outcomes, preventing misuse of prescription pain medications, and reducing the undertreatment of pain. In turn, better treatment of pain has the potential to also reduce the total costs of treatment. The focus of this paper is to identify and further explore the causes of the treatment gap in acute pain, highlighting the economic and clinical impacts of undertreatment. In the sections that follow, we discuss these dynamics, focusing on the challenges associated with the use of opioids in first-line treatment of acute pain.

## 2 Demand

The demand for pain treatment in the U.S. and globally has been increasing, primarily for three reasons: (1) an increase in the total number and rate of surgical procedures; (2) an increase in the proportion of the population over the age of 65, along with a lengthening of life expectancy; and (3) an increase in the prevalence of metabolic diseases, such as obesity and diabetes.

There has been an increase in the overall volume of surgical procedures. Apart from a slight dip during the COVID-19 pandemic, rates of surgeries in the U.S. have been steadily increasing for decades (9). This is especially the case for orthopedic procedures, where in the two-decade period from 2000 to 2019, the estimated annual volume of total hip arthroplasty (THA) and total knee arthroplasty (TKA) increased by 177% and 156%, respectively (10). In addition to increased demand for pain management associated with surgical volume, concomitant trends toward shorter lengths of hospital stays and migration of care to outpatient settings has further magnified demand for pain treatment (11, 12).

In addition, the proportion of the U.S. population that is aged 65 and over has grown steadily over the past several decades, and is expected to more than double by 2040 (13). Increased aging of the population, increased life expectancy, and improved survival rates from cancer and other chronic diseases has added further demands on the health system to develop more effective means of controlling acute and chronic pain for a growing number of individuals who are living longer with acute and chronic health problems (14, 15). Among the most worrisome trends in the prevalence of chronic health problems has been the steep growth in prevalence of metabolic diseases, such as obesity and diabetes, both of which are associated with increased prevalence of acute pain. Beyond the impact on acute pain, research has also shown that metabolic disorders, specifically those involving abnormal glucose metabolism, have been identified as significant risk factors for the development and persistence of chronic pain (16, 17). For example, Mäntyselkä et al. observed that diabetes mellitus and elevated plasma glucose were associated with daily chronic pain, increasing the risk of chronic pain in adults by almost 2.5 times (17).

From 2000 to 2018, obesity prevalence increased an unprecedented 39%, and rates of severe obesity increased an alarming 96% over the same time period (18). The connection between obesity and pain is both direct and indirect. The direct effect has been observed in studies of reported pain by individuals with varying degrees of obesity. For example, Hitt et al. found that Class II obese respondents (i.e., those with BMI of 35 to 39.9) were 1.9 times more likely to report severe pain, and Class III obese individuals were 2.3 times more likely to report severe pain (19). Although the causal pathways between obesity and chronic pain remains unclear, as a result of complex and bidirectional mechanisms, evidence has shown that individuals with obesity can have up to 45% higher risk of chronic pain compared to those of normal weight (20). This may be due in part to the fact that obesity can promote insulin resistance and accelerate the progression of peripheral neuropathy (21). The mechanisms associated with obesity also impact the overall burden and undertreatment of acute pain. Individuals with obesity demonstrated more than double the odds of intra-procedural acute pain compared to those without obesity (22). Additionally, pain management remains a challenge within this population, as the use of opioids, particularly among those with obstructive sleep apnea, is associated with increased risk of opioid-induced central sleep apnea and respiratory depression, which can result in higher mortality (23). These safety concerns often limit pain management, potentially further contributing to the undertreatment of acute pain.

The second pathway linking obesity and demand for pain treatment is indirect, as obesity is causative of a variety of serious health conditions that increase the need for medical care services, including pain management. Generally, these conditions include, but are not limited to, coronary artery disease, heart failure, cardiac arrhythmia, stroke, insulin resistance, diabetes mellitus, hypertension, sleep apnea, arthritis, musculoskeletal conditions, and certain types of cancer (24–27). Diabetes, in particular, is projected to affect 10.9% of the global population by 2045, an increase from 9.3% in 2019, with significant implications for pain management (28). Diabetes complications, such as neuropathy and poor wound healing, contribute to both acute and chronic pain, complicating treatment (29). In addition, surgical interventions related to diabetes, such as amputations, often result in severe pain, further driving the demand for pain management interventions. Growth in metabolic diseases has largely overlapped with the aging of the population, the cumulative effect of which has been a marked rise in the demand for pain management (30); since 1998, pain prevalence in U.S. adults has increased more than 25% (1).

## 3 Treatment

There are several treatments and approaches aimed at the management of acute pain. These approaches are generally categorized as non-pharmacologic and pharmacologic treatments. Non-pharmacological treatments include, for example, physical therapy, biofeedback, cognitive behavioral therapy, and massage therapy. While these types of treatments have been shown to be effective in some instances, their efficacy is generally limited to mild to moderate pain and by lower rates of adherence and barriers to access (31). Pharmacologic treatments are typically partitioned into opioid vs. non-opioid drugs such as acetaminophen and non-steroidal anti-inflammatory drugs (NSAIDs). In practice, optimal pain management frequently relies on a multimodal approach that combines these therapies. For procedures such as total knee arthroplasty, peripheral nerve blocks are commonly used to manage postoperative pain; however, protocols like those at Brigham and Women's Hospital (BWH) indicate that these blocks are typically administered either as a one-time dose or as a continuous infusion for only a brief period following surgery, often discontinued by the morning of the first postoperative day (32). Therefore, while opioids are generally reserved for moderate to severe pain, they are often relied upon for longer-term pain management following a procedure, as there have been no analgesics superior or as effective as opioids.

### 3.1 Serious adverse events

There is understandable trepidation regarding the use of opioids for pain. The serious adverse events (SAEs) and adverse drug reactions (ADRs) associated with opioids have been broadly discussed in the literature, increasingly in recent years due to the opioid crisis and the revised CPGs regarding acute pain. As current pain treatment guidelines generally acknowledge, despite their efficacy, opioids are associated with several important SAEs, notably physical dependence, euphoria, risk of overdose, and respiratory depression, which together can lead to significant negative outcomes. In addition, opioids are also associated with substantial humanistic and economic burden, mainly because they are linked to greater risk of misuse and diversion, both of which have the potential to magnify the risks associated with dependence and overdose.

**Figure 1:**
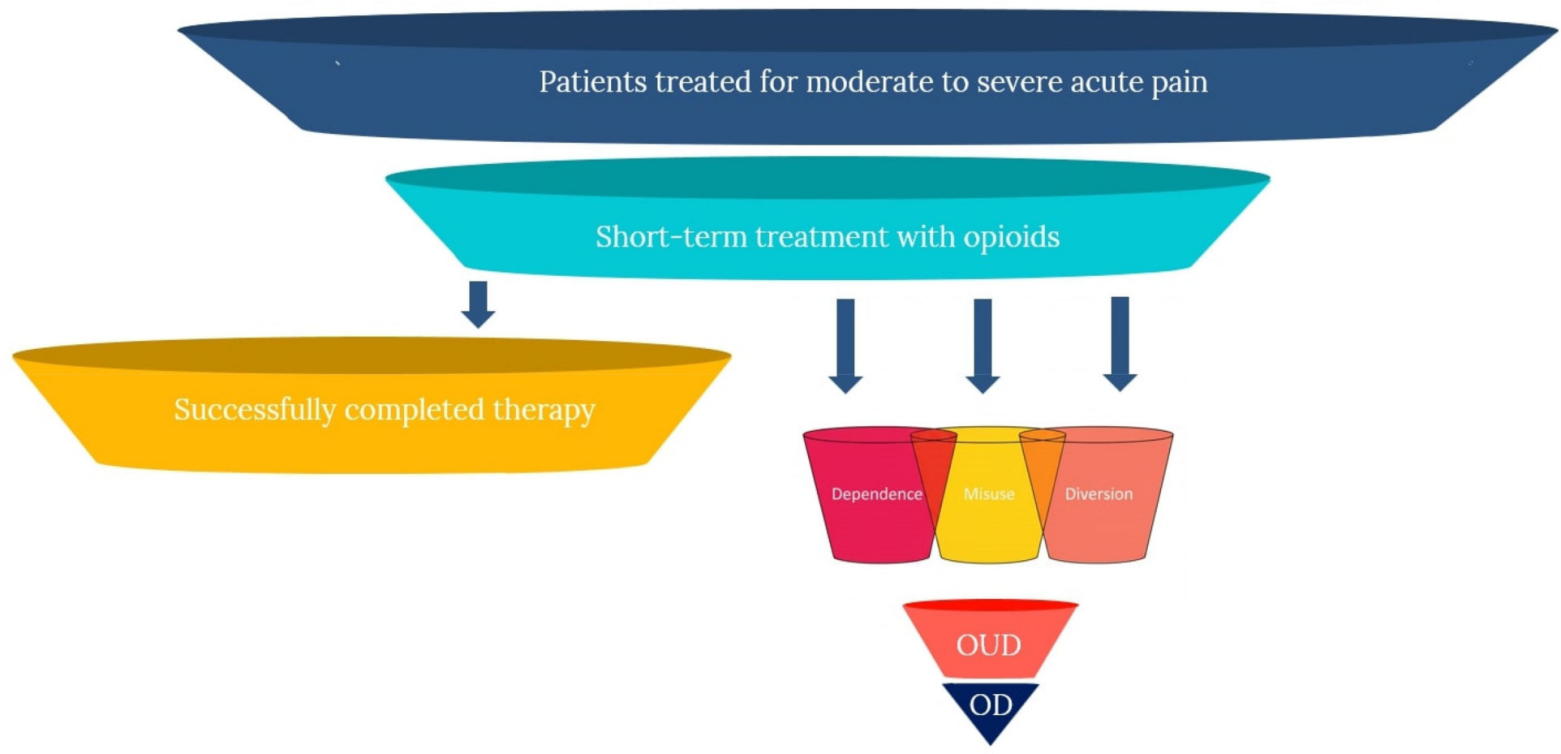
Patient pathways from acute pain treatment with opioids to outcomes.

As such, opioids are also associated with what economists refer to as “negative externalities,” where the negative effects of consumption extend beyond proximal clinical effects (i.e., SAEs and ADRs), and have the potential of generating substantial “secondary” harms in the community. Accordingly, in the case of opioids, it is more appropriate to consider the tradeoffs and limitations more broadly, as inclusive of these primary and secondary effects, resulting in the following five domains of negative effects: (1) risk of dependence; (2) risk of misuse; (3) risk of progression to use disorders; (4) risk of overdose; and (5) risk of diversion and secondary harms. The figure below illustrates the potential patient pathways following short-term opioid treatment for acute pain, demonstrating both successful resolution and progression to various harms. These effects are portrayed in Figure 1 below.

### 3.2 Dependence

The risk of physical dependence following treatment with opioids is an important clinical consideration and has also been a source of trepidation among patients considering opioid-based treatment approaches (33, 34). Opioid dependence develops in 11%–13% of patients initially prescribed opioids (35, 36). Schroeder et al., for example, in a matched case control study of opioid-naive adolescents and young adults, found that index opioid prescriptions were associated with a 6.8% absolute risk increase in persistent opioid use after dental procedures, which are usually associated with relatively short duration of pain (37). Moreover, opioid dependence can occur over a relatively short period of time, with some studies showing dependence occurring as soon as five days after an opioid prescription is obtained (38), and in some cases even after the patient no longer reports experiencing pain symptoms (39). This suggests that opioid use may continue beyond the need for pain relief, but instead due to emerging dependence.

In a large study of opioid-naive patients (surgical and non-surgical) all of the surgical procedures were associated with an increased risk of chronic opioid use, with odds ratios ranging from 1.28 for cesarean delivery to 5.10 for TKA (40). In another large study of opioid-naive patients undergoing short-stay surgery, Alam et al. found that patients receiving an opioid prescription within 7 days post-surgery were 44% more likely to become long-term opioid users within one year, compared to controls (41). Similar patterns have been observed among hospital discharges more generally; for example, Calcaterra et al. found that opioid receipt within 72 h of discharge was associated with increased likelihood of chronic opioid use and greater subsequent opioid refills one year post-discharge, compared to controls (42). Discontinuing therapy or reducing dosage, even when tapered per FDA approved prescribing information and medical guidelines, has been shown to be challenging and has a relatively high likelihood of failure (43, 44). Often tapering results in opioid use lasting significantly longer than necessary to alleviate symptoms (44), and tapering has also been shown to increase the probability of ED visits and hospitalizations (45).

### 3.3 Misuse

Data from the 2021 National Survey on Drug Use and Health (NSDUH) showed that, of the 68.5 million (27.0%) U.S. civilian adults who used prescription opioids, 8.3 million (12.1%) misused them (46). However, studies of misuse have shown that rates can vary substantially depending on population, methods, and timeframe, with some estimates of the prevalence of misuse as high as 29% (47). Apart from the aforementioned elevated risk of dependence and addiction, opioids are particularly prone to misuse because they cause euphoria, often even at doses needed for analgesia (48). This is the driving factor contributing to opioid misuse; often patients receive an initial prescription, experience feelings of euphoria, and then are induced to subsequently misuse, in many cases after pain has subsided.

Studies have shown that the experience of euphoria plays a significant role in driving the desire to continue using opioids beyond the point of medical need (49, 50). In a study of emergency department patients treated for pain with opioids, 25% reported experiencing pain relief in combination with sensations of being “high” and feelings of euphoria (51). In another study of ED patients with acute pain, patients randomized to opioids were significantly more likely to report that the medications were associated with feelings of “blissfulness.” (50) This euphoric sensation can trigger drug-seeking behavior, leading patients to misuse the medication in an attempt to recreate the euphoric feeling (52, 53). In turn, opioid seeking behavior can result in individuals cycling among euphoria, withdrawal, and craving, a process that is associated with increasing rates of misuse (54, 55) and, ultimately, poor treatment outcomes (56, 57). These effects have been shown to be dose dependent. For example, in a large study of commercially insured patients, Brat et al. found that total duration of opioid use was a strong predictor of misuse, with each refill and additional week of opioid use associated with an adjusted increase in the rate of misuse of 44% (58).

The risk profile for opioids also includes factors that transcend the traditional concepts of SAEs and ADRs. One of the most important of these differences is the propensity for prescription opioids to be “diverted” for non-medical use. Several studies have closely examined the sources of misused opioids. One of the most comprehensive of these studies is by Park and Wu, who analyzed data from the NSDUH (59). Across all age and sex categories, the authors found that 36.6% of individuals who reported opioid misuse during the past year obtained the opioids directly from the health system. The largest fraction (40.3%) obtained opioids unlawfully from friends and family members, and another 23.1% obtained them unlawfully from a variety of other sources, including drug dealers. These results are broadly consistent with other studies. For example, Schepis et al., in two different studies, found very consistent results within similar age groups (60, 61).

### 3.4 Progression to use disorder

The cycle of acute pain, opioid dependence, addiction, misuse, and poorly managed pain leaves individuals at substantially higher risk for progression to substance use disorders. The term “opioid use disorder” (OUD), which is listed in the American Psychiatric Association DSM-5, generally refers to repeated or consistent misuse of opioids, and includes more clinically serious forms of abuse or addiction (62). According to estimates based on the 2021 NSDUH, among US adults with past-year prescription opioid use, 4.8 million (7.0%) had prescription opioid use disorder (46). Similar to estimates of rates of misuse, rates of OUD vary depending on population, methods, and timeframe, with some estimates of OUD as high as 12% (47). Prescription opioids can in some cases serve as a “gateway” to non-medical use and progression to OUD (63). For example, Butler et al. found that among ED patients reporting nonmedical opioid use or heroin use, 59% indicated initial exposure via prescription opioids (64). A large study of dental patients conducted by Schroeder et al. found that 5.8% of opioid-exposed individuals experienced one or more subsequent health care encounters associated with an opioid abuse-related diagnosis, compared with 0.4% in the control group (37). These effects have also been shown to be dose dependent. In a large study of adolescents and young adults with opioid prescription claims in a large commercial claims database, Chua et al. found that each increase in daily opioid dosage category was associated with higher overdose risk [adjusted odds ratio (AOR), 1.18; 95% CI, 1.05–1.31] (65). In some cases prescription opioids can also act as a gateway to abuse of other more potent forms of opioids, including heroin and synthetic fentanyl, as well as progression to other forms of SUD (66, 67).

### 3.5 Overdose

While most definitions of overdose associated it with “excessive use,” more recent definitions have focused instead on physiology; for example, “a condition or physical state produced by the ingestion, injection, inhalation of or exposure to a deleterious agent.” (68). Individuals who use opioids are at increased risk for drug overdose (69, 70). Opioid overdose can include a mix of severe reactions, including hypoxia, respiratory distress, peripheral vasodilatation and hypotension, nausea and vomiting, psychiatric distress (e.g., anxiety, agitation, and dysphoria), and seizures (71).

The risk of overdose is particularly high among individuals who misuse opioids, for several reasons. First, those who misuse may take higher doses due to euphoria-seeking behavior (72). Second, misuse often progresses to non-oral administration as a means of obtaining a more rapid onset of effects (73), thereby giving the body less time to adapt to the respiratory depressant effects and increasing the risk of overdose. Opioid overdose is typically accompanied by respiratory depression, which is characterized by life-threatening changes in breathing frequency and oxygen saturation (74, 75). This risk is substantially greater in patients who are concomitantly prescribed other drugs that have the potential to inhibit respiratory function, such as benzodiazepines, gabapentin/pregabalin, first generation antihistamines, and tranquilizers (76). These drugs are often prescribed alongside opioids (77, 78). For example, individuals experiencing acute or chronic pain are more likely to also experience anxiety, and are therefore more likely to be prescribed benzodiazepines (79). Similarly, individuals experiencing acute or chronic pain are also more likely to be prescribed other pain medications, such as gabapentin (80, 81). First-generation antihistamines are also often used alongside opioids, typically to alleviate opioid side effects, such as pruritis and nausea (82).

These co-prescribed treatments have been shown to affect respiratory function additively and synergistically with opioids (77, 78, 83). For example, in 2016, the FDA issued its strongest warning against prescribing benzodiazepines alongside opioids due to evidence of increased risk of overdose and mortality (84). Similar concerns have been raised regarding opioid use concurrently with gabapentin and pregabalin (85–87). In addition, alcohol can potentiate opioid-induced respiratory depression (88). The risk of overdose is significantly greater in patients with underlying respiratory conditions, such as sleep apnea or asthma, and in patients with renal or hepatic impairment, as those conditions can significantly increase exposure to opioids (89–91). For example, Boitor et al. found that patients diagnosed with renal failure were more than two times more likely to experience opioid-induced respiratory depression compared to those without diagnosed renal failure (83). Often, renal and hepatic impairment is undiagnosed or can be acute due to infection or adverse effects of other drugs, thereby increasing risk of opioid-induced respiratory depression. Moreover, tolerance to opioid effects can develop rapidly, causing the need to increase dose levels; however, tolerance to the respiratory effects develops more slowly (92). Thus, as dosage increases, individuals with these comorbid conditions are at substantially increased risk of overdose.

In addition to co-prescribing with other drugs that can suppress respiratory function, opioid use disorder is also associated with “polysubstance use” (PSU), which is generally defined as the misuse of more than one drug at the same time or over a relatively short period (93). In 2019, among all deaths attributable to some form of drug overdose, nearly 50% involved observable PSU (93). Similarly, most opioid deaths involve PSU (94). For example, Morley et al. analyzed data from the Global Drug Survey and found that misuse of illicit drugs was associated with a *four-fold* greater likelihood of opioid misuse (95). Consequently, the risk of overdose and respiratory depression increases significantly when opioid use or misuse is accompanied by PSU, which can include non-medical use of the aforementioned co-prescribed drugs (especially benzodiazepines and tranquilizers), alcohol, cocaine, and other illicit drugs. The added risk associated with PSU is evident in much of the data on opioid mortality (96).

## 4 Treatment gaps

Substantial gaps in acute pain treatment persist with the rise in prevalence of acute pain, despite the availability of effective treatments, (30). For example, in a study of patients at a tertiary care hospital, about 50% of patients reported that their pain had not been relieved (97). Another study found that only 60% of ED patients received pain medications, but 74% continued to report moderate to severe pain upon discharge (98). Similarly, a large national survey found that more than 80% of patients experienced acute post-surgical pain within two weeks of discharge, and most of those patients indicated that their pain was moderate to severe (99).

### 4.1 Opioids

In many cases, moderate to severe acute pain can only effectively be treated with opioids (100–102). As mentioned previously, no other analgesics have demonstrated superior or even similar efficacy to opioids for the treatment of nociceptive pain. However, despite their clinical effectiveness, the use of opioids is, understandably, associated with considerable apprehension, for many of the reasons discussed in the previous section. Apprehension regarding opioid use to treat moderate to severe acute pain is generally attributable to four factors: (1) uncertainty on the part of providers to prescribe opioids, due to challenges in assessing risk-benefit tradeoffs and perceptions regarding opioid prescribing guidelines; (2) uncertainty on the part of patients, who also experience challenges understanding risk-benefit tradeoffs; (3) increased prevalence of state-level policies, which have either substantially restricted access to opioids or generated uncertainty regarding appropriate use and access to opioids; and (4) underappreciation of the long-term consequences of ineffectively treating acute pain. However, given the relatively recent prominence of opioid information in the medical literature and mainstream media, especially regarding the crisis of addiction and overdose, the ascension in the importance of these factors is not particularly surprising. More importantly, these factors have direct medical consequences, because each one of the factors has the potential to impede access to effective management of acute pain.

### 4.2 Providers

Uncertainty and reluctance on the part of providers to prescribe opioids has been a substantial impediment to the management of acute and chronic pain (30). Provider reluctance and uncertainty regarding opioids intensified during the opioid crisis, with some providers expressing general concerns regarding dependence, misuse, addiction, and diversion (103–106). In a recent comprehensive review of the literature on physician attitudes toward opioid prescribing, Bell et al. identified five common themes regarding opioid prescribing challenges: (1) uncertainty stemming from the subjectivity of pain; (2) concern regarding patient SAEs and community effects, such as diversion; (3) previous negative experiences, such as negative professional feedback and patient feedback; (4) uncertainty and confusion regarding interpretation and implementation of CPGs; and (5) conflicting institutional policies and burdensome administrative requirements (107). More generally, another contributing factor leading to gaps in acute pain management has been lack of consistency on the part of providers in following acute pain CPGs. For example, a recent systematic review identified 20 pain management quality measures, but only three of which focused specifically on postoperative pain management, leading the authors to conclude that “these results indicate a lack of measures to guide and promote quality postoperative pain management.” (108). Again, given the high level of attention garnered by the crisis of addiction and overdose, these reactions on the part of providers have been understandable. Perhaps more importantly, there exist no readily available solutions to provider reluctance and apprehension.

### 4.3 Patients

Many of the same factors affecting provider prescribing have also impacted patients (102). Fear of longer-term dependence and addiction are generally the main concerns expressed by patients (33). In addition, for some patients there is a perceived stigma associated with opioid use, and for these patients, opioid use can trigger feelings of shame, which can be exacerbated by uncertainty regarding risk of dependence and addition and discomfort in communicating concerns to their providers (109). Even patients who have been previously treated with opioids share some of these concerns; for example, Vargas-Schaffer et al. found that, in a sample of longer term opioid users, 40% maintained a “negative attitude” toward opioids, compared to 32% and 22% with positive and neutral attitudes, respectively (110). Again, reluctance on the part of patients is not unexpected given that many of their providers share the same reluctance.

### 4.4 Guidelines and policy

In addition to provider and patient factors, state and federal policy has impacted the use of opioids for moderate to severe acute pain. Prior to 2016, clinical practice guidelines (CPGs) for the treatment of acute pain generally considered opioids as first-line treatment, primarily due to better efficacy relative to alternative treatments (102, 111, 112). In 2016, the U.S. Centers for Disease Control and Prevention (CDC) issued opioid prescribing guidelines in response to increasing rates of opioid dependency and overdose, with the main thrust of the guidelines aimed at encouraging greater caution in prescribing of opioids for acute and chronic pain (113). However, the CDC guidelines were by most accounts “over interpreted,” resulting in the exacerbation of many of the problems that prompted opioid prescribing initially; that is, the management of the escalating prevalence of acute and chronic pain (114). Specifically, the CDC guidelines resulted in an increased propensity for providers to be overly rigid regarding dosage, duration, tapering, and discontinuation, and to misapply the guidelines to populations outside of the guideline scope (e.g., the misapplication of chronic pain recommendations to acute pain management) (115). Moreover, following the original 2016 CDC guidelines, one study found that only two thirds of opioid prescribing physicians were even aware of the existence of the guideline (116). While no current study directly links the introduction of recent CDC opioid prescribing guidelines to increased rates of chronic postsurgical pain, there is strong and consistent evidence in the literature that inadequate management of acute pain, especially during the postoperative period, significantly increases the risk of chronic pain (117–121). For example, Zhang et al. specifically examined how mechanisms across multiple domains such as epigenetic changes, including DNA methylation and histone modifications that alter the expression of pain-related genes, can contribute to the development of chronic pain, emphasizing that the timing of intervention is crucial for disrupting this progression and preventing pain chronification (121).

Some believed that the CDC guidelines were either unclear or advocated for excessive levels of caution, prompting the CDC to release revised guidelines in 2022 (122). However, the main objective of the 2022 revisions was largely the same, encouraging providers to exercise extreme caution regarding opioid utilization, and consider opioid treatments only in cases where non-opioid treatments have failed or where the likelihood of SAEs precluded non-opioid options. Although much of the focus of the 2016 CDC opioid guidelines was on the overuse of opioids in the treatment of chronic pain, the guidelines also addressed acute pain by recommending limiting opioid use for acute pain to 3–7 days (113). Professional medical societies generally followed the CDC guidance in issuing their own acute pain treatment CPGs (123–127).

Following the CDC guidelines, many states in the U.S. began to implement their own restrictions and recommendations. As of 2021, 29 states had some kind of limitation on the duration of opioid prescriptions, with 15 states imposing moderate restrictions, such as limiting initial prescriptions to 7 days. Three states restricted prescriptions to a 30-day supply, and 10 states had more stringent limits, including restrictions based on dosage. States like West Virginia, Kentucky, and Florida have implemented some of the more restrictive measures (128). Overall, these policies have been shown to have resulted in substantial reductions in opioid prescribing (129). Moreover, at the federal level, Congress recently introduced H.R. 5172, the “Non-Opioids Prevent Addiction in the Nation Act,” which is conventionally known as the “NO PAIN Act.” The Act would essentially increase financial incentives, via reimbursement mechanisms, to prescribe non-opioid treatments. In addition, heightened scrutiny by the U.S. Drug Enforcement Administration (DEA) regarding opioid manufacturing, shipments and utilization (130). The burden of acute pain may be exacerbated with the focus of these guidelines and policies shifting from the treatment of pain to the mitigation of treatment side effects, in light of the current treatment options.

### 4.5 Pain progression

The consequences of insufficient treatment of acute pain extend beyond short-term patient comfort. A particularly important issue that leads to significant patient clinical and economic burden is the progression of poorly treated acute pain to chronic pain, sometimes referred to the “chronification” of acute pain (117, 118, 131–136). There is evidence of this process occurring, with the proportion of post-surgical patients developing chronic pain (i.e., chronic post-surgical pain, or CPSP) in the range of 10%–40%, depending on the type of surgery (137, 138). Hip arthroplasty, for example, has been associated with a 27% risk of CPSP, and knee arthroplasty associated with a 13%−44% risk of CPSP (138). In addition to chronic post-surgical pain, there is also evidence of chronification of acute pain following ED visits. For example, in a study of ED patients, those who departed the ED with a high pain score (e.g., 7/10 or higher) were significantly more likely to develop chronic pain within 90 days (139). In addition, another long-term negative outcome of ineffective pain treatment and resulting chronification is that it has been shown to increase the likelihood of dementia in the elderly (140–142).

### 4.6 Economic impact

Despite the increase in the prevalence of moderate to severe acute pain, opioid prescriptions have fallen dramatically over the past decade, particularly after the release of the CDC guidelines and the implementation of state policies restricting opioid use (143). After a period of steadily increasing rates from the late 1990s until around 2012, opioid prescription rates began to steadily fall and continued to fall through 2020, from about 250 million prescriptions in 2012 to less than 150 million prescriptions in 2020 (144). Due to apprehensions regarding opioids, over that time period, opioids have been largely replaced by less effective non-opioid options.

The high prevalence of pain, the undertreatment of acute and chronic pain, and the progression of undertreated pain is associated with significant economic burden. The attributable costs of pain, including pain treatment, have been found to vary considerably depending on causal pathways and type and intensity of treatment. Some broad estimates were developed by Gaskin and Richard, who estimated the total costs of pain (acute and chronic) in the U.S. using data from the Medical Expenditure Panel Survey (MEPS), finding that the attributable “excess” healthcare costs of pain summed to nearly $436 billion (in 2024 dollars) (145).^1^ The impact of pain on work productivity, as measured by absenteeism, presenteeism, and depressed wages, added another $487 billion, bringing the total attributable cost of pain to $923 billion (in 2024 dollars). Their analysis also found that moderate pain doubled healthcare costs, whereas severe pain tripled these costs. Notably, these costs, which were based on 2010 data, are likely an underestimate of total attributable pain costs in more recent years, mainly due to the increasing prevalence of acute pain and the rapid growth of interventional pain techniques, the utilization of which increased more than two-fold from 2000 to 2013. (146) Private insurers bear the largest proportion of healthcare costs attributable to pain (43%) (147).

Moreover, in addition to these costs are the many indirect expenses tied to the use of opioids for pain management, including the long-term impact of opioid use disorder, misuse, and overdose. In 2017 alone, the total economic burden of OUD and opioid-related fatal overdoses in the U.S. was approximately $1.02 trillion, including $92 billion from lost productivity and $23 billion from criminal justice-related expenditures (148). As previously discussed, many physicians have historically opted to treat pain with opioids; however, the substantial healthcare and societal costs associated with opioid use disorder and overdoses indicate that it may be time to adopt alternative, non-opioid approaches to pain management. The rising indirect and societal costs mentioned above has partially played a role in shifts in opioid prescribing practices.

## 5 Discussion

The challenge of managing acute pain with opioids is a difficult conundrum, as both prescribing and not prescribing opioids present significant risks. On one hand, opioid use for acute pain poses significant risks for adverse effects, such as dependence, misuse, OUD, overdose, and increased healthcare utilization and costs. Patients who are prescribed opioids postoperatively or for acute pain often experience higher rates of persistent opioid use, which in turn leads to more ED visits, hospitalizations, and the need for treatment. Moreover, opioids prescribed to individuals with pain are frequently diverted to others who misuse them. While opioids are effective at managing severe to moderate pain, the risk of associated adverse events and potential for misuse make it a challenging option.

On the other hand, withholding or restricting prescribing opioids due to these risks can lead to undertreatment of acute pain, which itself has serious consequences. The undertreatment of acute pain can result in the chronification of pain. Patients who do not receive appropriate pain management are more likely to suffer from poor recovery, worse outcomes, and long-term disability, resulting in diminished quality of life. The fear of OUD and the apprehension surrounding opioid use have led to more conservative prescribing practices by physicians and patients often refusing treatment, leaving some patients undertreated. This situation highlights the need for innovative pain management alternatives that are as effective as opioids but without the associated risks of dependence, misuse, progression to use disorders, and overdose. The healthcare system also faces systematic barriers, such as practice guidelines and state and federal regulations that restrict the prescription of opioids even for patients for whom the risk-benefit tradeoff favor prescribing. Consequently, these restrictions, intended to curb misuse and address the ongoing opioid crisis, often result in the undertreatment of patients.

## 6 Conclusions

The current approach to managing acute pain continues to be a difficult balancing act. The use of opioids, while often necessary to effectively manage acute pain, carries significant risks, including dependence, misuse, OUD, and overdose. On the other hand, not treating pain adequately can lead to worse short-term outcomes, the chronification of acute pain, and poor long-term outcomes. As a result, there is compelling demand for new and innovative pain management options that offer the efficacy of opioids without the associated risks. When such options are available, it is important that guidelines are reformed in order to appropriately treat pain while also minimizing harm and preventing opioid misuse, especially as we continue to address the opioid crisis.

## Footnotes

^1^Data reported by authors in 2010 dollars, trended forward to 2024 using the U.S. Consumer Price Index (“CPI”).
